# Deep Learning-Based Image Quality Enhancement Combining Denoising and Super-Resolution for Fat-Suppressed T2-Weighted Breast MRI: A Qualitative and Quantitative Evaluation

**DOI:** 10.7759/cureus.100771

**Published:** 2026-01-04

**Authors:** Tatsuya Hayashi, Shizuho Shikama, Yumi Ibaraki, Shinya Kojima, Shimpei Yano, Tomoyuki Fujioka, Hiroshi Oba

**Affiliations:** 1 Graduate School of Medical Technology, Teikyo University, Tokyo, JPN; 2 Central Radiology Division, Teikyo University Hospital, Tokyo, JPN; 3 Department of Radiology, Dokkyo Medical University Saitama Medical Center, Saitama, JPN; 4 Department of Radiology, Teikyo University School of Medicine, Tokyo, JPN

**Keywords:** breast mri, contrast ratio, deep learning reconstruction, denoising, signal-to-noise ratio, super-resolution

## Abstract

Introduction

Fat-suppressed T2-weighted breast MRI faces a trade-off among signal-to-noise ratio (SNR), resolution, and scan time. This study evaluated the intrinsic effect of a commercial deep learning reconstruction (DLR) method, combining denoising and super-resolution, on image quality by comparing it with conventional reconstruction (Conv) generated from identical raw data.

Materials and methods

For this retrospective study, 49 women who underwent 3-T breast MRI were included. From the same k-space data, Conv and DLR images were produced. Qualitative assessment involved two blinded readers qualitatively scoring five image quality parameters. For quantitative analysis (n = 44 analyzable cases), regions of interest were placed to define the SNR within the pectoralis major and the contrast ratio (CR) between muscle and fat.

Results

Qualitatively, DLR yielded higher scores for contrast, noise, and depiction of breast parenchyma for both readers (all p < 0.001). Signal uniformity improved modestly for one reader. Artifact ratings were mixed: one reader favored DLR (p = 0.002), whereas the other showed no significant difference (p = 0.670). Inter-reader agreement was good to very good for most parameters (kappa = 0.75-0.83), but moderate for artifacts (kappa = 0.42). Quantitatively, DLR increased the SNR by approximately 31% (median 5.00 vs. 3.79; p < 0.001), while the CR changed minimally (median 0.56 vs. 0.53; p = 0.029).

Conclusion

These findings indicate that DLR enhances perceived conspicuity and SNR via denoising and sharpening while preserving intrinsic tissue contrast. Applying DLR without altering acquisition parameters intrinsically improves image quality, supporting future protocol optimization toward shorter scan times or higher resolution.

## Introduction

Deep learning reconstruction (DLR) has emerged as an innovative approach to address the challenges of long acquisition times and noise in magnetic resonance imaging (MRI) [1,2]. DLR employs convolutional neural networks trained on large image datasets to suppress noise and enhance spatial resolution during or after reconstruction, typically using k-space or image-domain data as input, and can provide more effective noise suppression than conventional methods [1]. Furthermore, super-resolution processing enhances image sharpness, potentially overcoming the traditional trade-off among acquisition time, spatial resolution, and signal-to-noise ratio (SNR).

Previous studies have demonstrated image quality enhancements achieved through DLR, such as noise reduction and apparent improvements in spatial resolution, across various anatomic regions. In prostate MRI, DLR has been reported to improve image quality while reducing scan time by approximately 36%, and in knee MRI, scan time reductions of up to 41% have been achieved without compromising diagnostic performance [3,4]. In abdominal imaging, DLR has resulted in significant improvements in organ conspicuity and overall image quality compared with compressed sensing [5]. Additionally, in head and neck T2-weighted imaging, the implementation of DLR combining denoising and super-resolution has enabled reduced scan times while maintaining enhanced image quality and SNR [6]. DLR has also been applied to diffusion-weighted imaging, where it improves image quality, maintains apparent diffusion coefficient accuracy, and enhances measurement precision in phantom experiments [7,8]. Collectively, these findings underscore DLR as a versatile and robust reconstruction technique applicable across diverse body regions and imaging sequences.

Breast MRI is an indispensable imaging modality with high sensitivity that frequently outperforms other imaging techniques in screening women with dense breasts [9]. Although dynamic contrast-enhanced MRI (DCE-MRI) serves as the diagnostic cornerstone, T2-weighted imaging (T2WI) plays a crucial role in characterizing lesions with high T2 signal intensity, such as cysts, hemorrhage, and mucin, and complements DCE-MRI by improving specificity in differentiating benign from malignant lesions [10,11]. However, T2WI generally requires a long repetition time, which prolongs acquisition and makes images more susceptible to motion artifacts and subsequent quality degradation. In clinical practice, efforts to reduce scan time often involve trade-offs that compromise spatial resolution and SNR [12].

Although clinical studies on DLR in breast MRI remain limited, recent research has investigated its application to fat-suppressed T2WI to address these challenges. Fat-suppressed three-dimensional T2WI (3D-T2WI) has been shown to significantly improve the depiction of lesion morphology and tissue structure, as well as overall image quality and diagnostic information, compared with conventional two-dimensional T2WI (2D-T2WI) [13]. Furthermore, the application of vendor-provided DLR has demonstrated improvements in both quantitative and qualitative image quality through noise reduction and edge enhancement [12]. In a prospective study employing DLR that combined denoising and super-resolution for breast MRI, fast spin-echo (FSE) imaging with the Dixon method achieved substantial scan time reductions while improving SNR, contrast-to-noise ratio, visualization of fine lesion structures, and noise suppression [14]. However, these previous investigations were optimized for specific imaging conditions-such as 3D-dedicated protocols or the Dixon method-and thus do not directly reflect the performance of DLR on the conventional fat-suppressed 2D-T2WI protocols that still serve as workhorse sequences in many breast MRI practices. Because 3D-T2WI and Dixon-based techniques are not yet universally available or fully stabilized across all scanners and institutions, it remains clinically relevant to clarify how much image-quality improvement can be obtained when DLR is introduced into standard 2D acquisitions without changing the acquisition parameters.

Accordingly, the purpose of this study was to apply a commercially available DLR method that simultaneously combines denoising and super-resolution to fat-suppressed T2-weighted breast MRI and to evaluate its effect on image quality through both qualitative and quantitative assessments.

## Materials and methods

Study design and patients

This single-center, retrospective observational study was approved by the institutional review board (approval No. 24-068). Owing to its retrospective nature, the requirement for informed consent was waived. A total of 49 consecutive women who underwent clinically indicated bilateral breast MRI at our hospital between March 2024 and July 2024 using the standard protocol that incorporates axial fat-suppressed 2D T2WI were included. No additional exclusion criteria were applied, and no examinations were excluded after screening. The mean patient age was 56 years (range: 32-90 years).

MRI acquisition and image reconstruction

**Figure 1:**
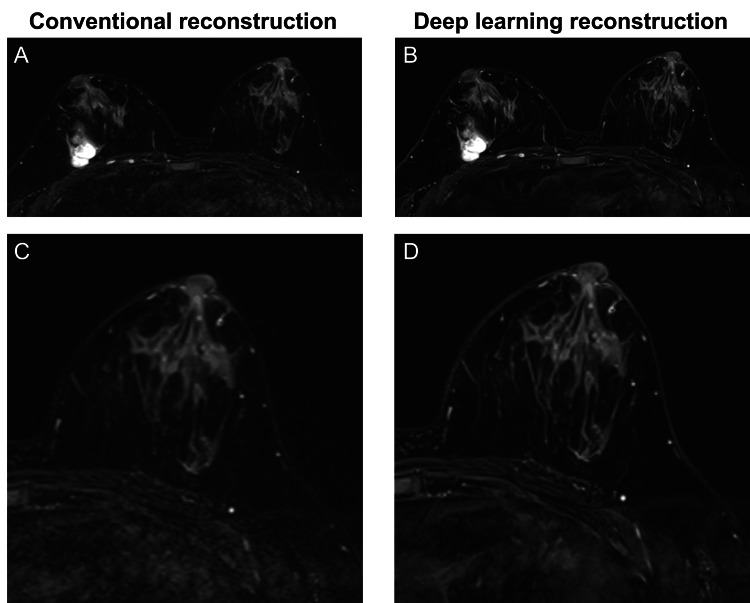
Conventional versus deep learning reconstruction on fat-suppressed T2-weighted breast MRI Images were reconstructed using the conventional method (A) and deep learning reconstruction (B). Panels C and D present magnified views of the right breast corresponding to A and B, highlighting fine structures. Compared with conventional reconstruction, deep learning reconstruction demonstrates clearer edge definition and finer parenchymal structures. All images were reconstructed from the same raw data using both the conventional and deep learning methods.

All MRI examinations were performed using a 3-T scanner (MAGNETOM Vida Fit, Siemens Healthineers, Erlangen, Germany) equipped with a dedicated 18-channel breast coil. Axial fat-suppressed T2-weighted images were acquired using an FSE sequence, with fat suppression achieved through spectral adiabatic inversion recovery. From the same k-space raw data, conventional reconstruction (Conv) and DLR images were generated separately (Figure 1). Conv images were reconstructed using the standard vendor-supplied 2D FSE reconstruction. The imaging parameters were as follows: field of view, 360 mm; acquisition matrix, 480 × 480; reconstruction matrix, 960 × 960 (interpolation for Conv and super-resolution for DLR); slice thickness, 3.0 mm; interslice gap, 0.6 mm; repetition time (TR), 4220 ms; echo time (TE), 83 ms; parallel imaging with generalized autocalibrating partially parallel acquisitions using an acceleration factor of 4; number of acquisitions, 1; bandwidth, 306 Hz/pixel; and acquisition time, 2 min 15 s. The DLR algorithm applied in this study was a commercially available deep learning-based reconstruction method provided by the scanner manufacturer (Deep Resolve Boost and Deep Resolve Sharp, Siemens Healthineers, Erlangen, Germany). According to the manufacturer, Deep Resolve Sharp is a deep learning-based technique that uses a deep neural network trained on both low-resolution and high-resolution MR data so that high-resolution images can be reconstructed from low-resolution acquisitions. The reconstruction is checked against the acquired raw k-space data, which are also incorporated into the reconstruction process to maintain consistency and reproducibility. Deep Resolve Boost integrates deep neural networks into an iterative reconstruction process; by passing the data through multiple networks, it enables accurate noise reduction. The manufacturer further reports that the training data for Deep Resolve Boost include both non-parallel-imaging data and highly accelerated parallel-imaging data, which allows the use of very high parallel imaging acceleration factors in clinical scans. DLR images achieved higher spatial resolution through super-resolution processing, whereas Conv images used interpolation; the resulting high-resolution datasets were subsequently compared.

Qualitative image assessment

Two radiological technologists with 10 years of MRI experience independently performed a blinded qualitative assessment of image quality. For each case, paired DLR and Conv images were displayed in random order. The readers, blinded to the reconstruction type, rated image quality on a five-point Likert scale (1 = very poor, 2 = poor, 3 = fair, 4 = good, 5 = excellent) across the following five criteria: contrast (clarity of boundaries among glandular tissue, fat, and muscle), noise (degree of suppression of granular noise throughout the image), signal uniformity (consistency of signal intensity within fat-suppressed regions across both breasts), depiction of breast parenchyma (visibility of fine parenchymal structures), and artifacts (extent of motion-related artifacts).

Quantitative image assessment

**Figure 2:**
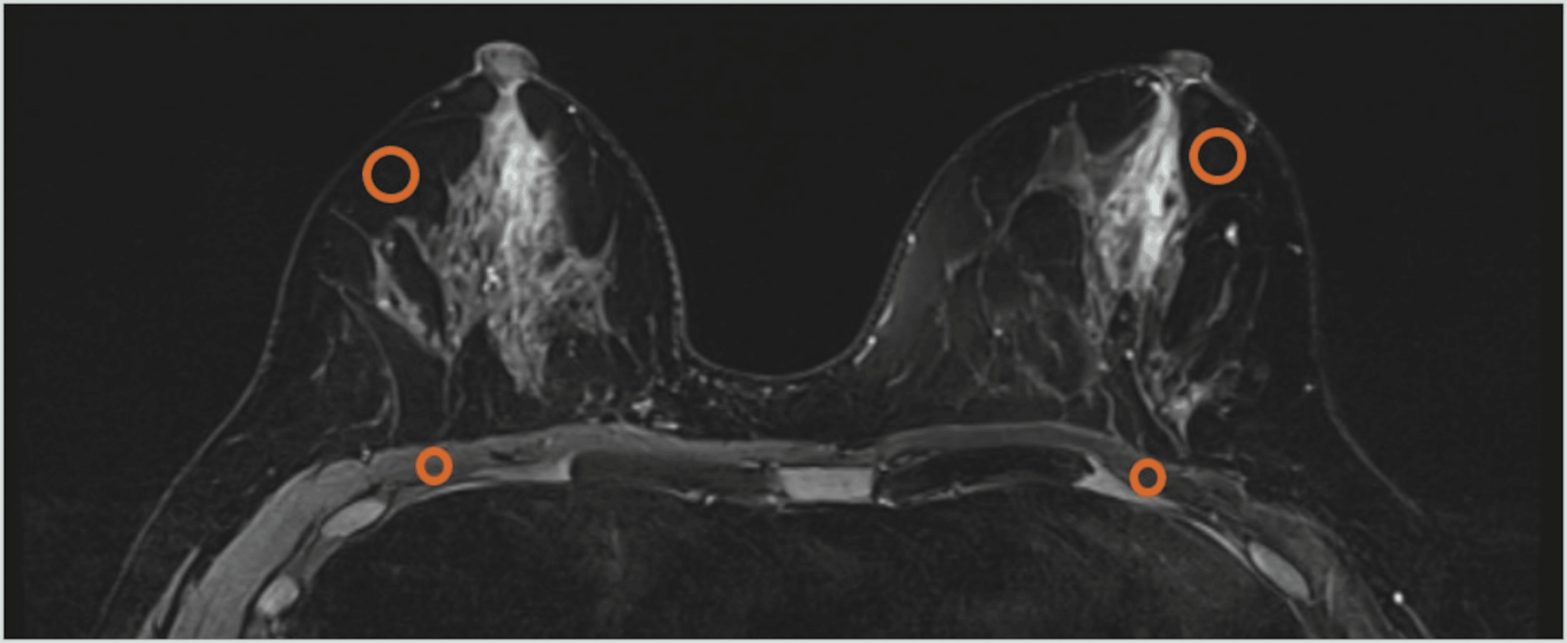
Representative placement of regions of interest for signal-to-noise ratio and contrast ratio on fat-suppressed T2-weighted breast MRI Orange circles indicate the circular regions of interest (ROIs). ROIs were placed within the bilateral pectoralis major muscles and within homogeneous fat regions in the fat-suppressed image. ROIs were positioned to avoid artifacts and large vessels and were copied to the corresponding locations on the paired reconstruction.

SNR and contrast ratio (CR) were calculated from the reconstructed images. Because denoising through DLR is a nonlinear process distinct from conventional linear filtering and can alter noise characteristics, traditional SNR estimation based on the standard deviation of a background region of interest (ROI) may be unreliable in this context [4]. Therefore, SNR was defined as the mean signal intensity within an ROI divided by the standard deviation within the same ROI on each image. Circular ROIs, each containing 448 pixels, were placed for measurement. To account for interindividual variability in fibroglandular tissue distribution, SNR was measured in the bilateral pectoralis major muscles, and CR was measured between the pectoralis major muscle and a homogeneous fat-suppressed region within the breast [12]. ROIs were positioned to avoid artifacts and large blood vessels (Figure 2). The ROIs were then copied to corresponding locations on the paired reconstructions. SNR and CR were measured separately for the right and left breasts, and the average of both breasts was used as the representative value for each case. The formulas were as follows:

SNR = SI_muscle_ / SD_muscle_

CR = (SI_muscle_ − SI_fat_) / (SI_muscle_ + SI_fat_).

Here, SI_muscle_ and SD_muscle_ represent the mean and standard deviation of the signal intensity within the pectoralis major muscle ROI, respectively, and SI_fat_ represents the mean signal intensity within the fat ROI.

Statistical analysis

The normality of continuous variables was assessed using the Shapiro-Wilk test. Variables determined to be non-normally distributed were summarized as medians with interquartile ranges (IQRs). Comparisons of qualitative scores and quantitative parameters were performed using the Wilcoxon signed-rank test for paired data. To adjust for multiple comparisons, the Holm method (Holm-Bonferroni correction) was applied. Comparison families were defined as the five qualitative criteria within each reader and the quantitative parameters (SNR and CR), and Holm adjustment was performed within each family. Inter-reader agreement for qualitative assessments was evaluated using the weighted kappa (κ) coefficient. The κ values were interpreted as follows: < 0.20, poor; 0.21-0.40, fair; 0.41-0.60, moderate; 0.61-0.80, good; and 0.81-1.00, very good. A two-sided p < 0.05 was considered statistically significant. Ninety-five percent confidence intervals (95% CIs) were reported for all κ values. Statistical analyses were conducted using BellCurve for Excel (Social Survey Research Information Co., Ltd., Tokyo, Japan).

## Results

Qualitative image assessment

The results of the visual assessment by two readers in 49 patients are summarized in Table 1. For the three criteria, contrast, noise, and depiction of breast parenchyma, the DLR images received significantly higher scores than the Conv images for both readers (all p < 0.001). For signal uniformity, the DLR images demonstrated a small but significant improvement for one reader. Regarding artifacts, reader opinions diverged: Reader 1 favored DLR (median 4, IQR 3-4 vs. 3, IQR 2-3; p = 0.002), whereas Reader 2 showed no significant difference (median 3, IQR 3-4 vs. 3, IQR 3-4; p = 0.670).

**Table 1:** Comparison of qualitative image-quality scores Medians with brackets denote interquartile ranges (IQRs). Deep learning reconstruction and conventional reconstruction images were compared using the Wilcoxon signed-rank test. p-values are Holm-adjusted.

Item	Reader	Deep learning reconstruction	Conventional reconstruction	Z value	p-value
Contrast	1	5 [4–5]	3 [2–3]	6.34	<0.001
	2	5 [4–5]	2 [2–2]	6.15	<0.001
Noise	1	4 [4–5]	2 [2–3]	6.23	<0.001
	2	5 [4–5]	2 [1–2]	6.17	<0.001
Signal uniformity	1	4 [2–4]	3 [2–4]	1.97	0.049
	2	4 [3–5]	3 [3–5]	1.93	0.108
Depiction of breast parenchyma	1	4 [4–5]	3 [2–3]	6.21	<0.001
	2	4 [4–5]	2 [2–2]	6.19	<0.001
Artifacts	1	4 [3–4]	3 [2–3]	3.25	0.002
	2	3 [3–4]	3 [3–4]	0.43	0.670

Inter-reader agreement for the qualitative assessment was evaluated using the weighted kappa (κ) coefficient. Agreement was very good for contrast (κ = 0.81; 95% CI, 0.77-0.86) and noise (κ = 0.83; 95% CI, 0.80-0.86), good for signal uniformity (κ = 0.75; 95% CI, 0.70-0.79) and depiction of breast parenchyma (κ = 0.78; 95% CI, 0.73-0.83), and moderate for artifacts (κ = 0.42; 95% CI, 0.28-0.56).

Quantitative image assessment

Of the 49 cases, 5 were excluded from the quantitative assessment because the bilateral pectoralis major muscles were extremely thin, making ROI placement unreliable. The remaining 44 cases were included in the analysis. The quantitative results are presented in Table 2. The SNR of the DLR images (median 5.00, IQR 3.54-6.06) was approximately 31% higher than that of the Conv images (median 3.79, IQR 3.15-4.96), a difference that was statistically significant (p < 0.001). The CR of the DLR images (median 0.56, IQR 0.35-0.68) was also significantly higher than that of the Conv images (median 0.53, IQR 0.37-0.63; p = 0.029), although the median difference was small (0.03).

**Table 2:** Comparison of quantitative image-quality metrics Medians with brackets denote interquartile ranges (IQRs). p-values are Holm-adjusted. Deep learning reconstruction and conventional reconstruction images were compared using the Wilcoxon signed-rank test. SNR, signal-to-noise ratio; CR, contrast ratio.

Metric	Deep learning reconstruction	Conventional reconstruction	Z value	p-value
SNR (pectoralis major)	5.00 [3.54–6.06]	3.79 [3.15–4.96]	5.78	<0.001
CR (muscle–fat)	0.56 [0.35–0.68]	0.53 [0.37–0.63]	2.18	0.029

## Discussion

This study directly compared conventional reconstruction with deep learning reconstruction (DLR; Deep Resolve Boost + Deep Resolve Sharp) using identical raw data and demonstrated significant improvements in SNR and subjective conspicuity on fat-suppressed T2WI. Whereas previous studies employing 3D-T2WI and Dixon-based techniques evaluated the combined effects of protocol modification and DLR [13,14], the present study maintained fixed acquisition parameters and compared only the reconstruction methods, thereby isolating the intrinsic effect of DLR. An additional strength of our study is that the evaluated sequence represents a standard fat-suppressed 2D-T2WI protocol that is widely used in routine breast MRI, rather than a dedicated 3D or Dixon-based protocol. Applying DLR without changing the acquisition parameters of such a workhorse sequence makes the observed improvements directly transferable to everyday practice. In this sense, our results describe the baseline benefit that can be expected when DLR is introduced into existing 2D-T2WI protocols, and they complement prior reports that focused on optimized 3D or Dixon-based acquisitions. Although the quantitative increase in CR was minimal and of limited practical relevance, perceived contrast improved substantially owing to boundary enhancement and noise suppression. These findings indicate that DLR preserves intrinsic image contrast while enhancing perceived conspicuity through its integrated denoising and super-resolution processes. Although scan time reduction was not directly assessed, the consistent improvements in image quality metrics with DLR broaden protocol design possibilities, suggesting the feasibility of increasing spatial resolution or anatomical coverage without extending scan time, or alternatively, reducing scan time while maintaining spatial resolution.

In our cohort, the SNR increased by approximately 31% on a median basis (DLR, 5.00 vs. Conv, 3.79; p < 0.001), confirming a DLR-specific effect under identical acquisition conditions. Phantom studies have shown that applying DLR to 2D-T2WI can increase SNR by a factor of 1.2 to 2.8 compared with conventional reconstruction, and our observed improvement falls within this range [12]. In a study that shortened acquisitions specifically for DLR, the application of denoising and super-resolution DLR to a 3-T T2-weighted Dixon sequence increased fibroglandular SNR compared with conventional reconstruction (50.9 ± 26.1 vs. 40.1 ± 19.5) [14]. The magnitude of SNR improvement, however, depends on the measurement method, anatomical site, and sequence characteristics (e.g., two-dimensional vs. three-dimensional). Furthermore, because DLR modifies noise statistics through a nonlinear process, traditional SNR estimation based on the standard deviation of a background ROI may not accurately reflect intrinsic signal properties [4,15]. In this study, we employed the within-ROI standard deviation approach; however, this method is not absolutely definitive. Therefore, SNR and CR should be regarded as comparative indices. Accordingly, a multidimensional evaluation that integrates subjective image quality scores with quantitative metrics is essential for the comprehensive assessment of DLR images.

Whether DLR preserves native lesion-to-background contrast is a crucial consideration. In breast MRI, T2-weighted contrast facilitates differential diagnosis; for example, lesions with very high signal intensity on T2WI often represent benign entities such as cysts or fibroadenomas, whereas many malignant lesions exhibit intermediate signal intensity. Any alteration in lesion-to-background contrast could therefore affect diagnostic interpretation. In our data, the CR was significantly higher with DLR, but the median difference was only approximately 0.03, which is likely clinically negligible. Previous work comparing compressed-sensing 3D-T2WI reconstructed with and without DLR demonstrated a small, statistically nonsignificant increase in CR with DLR (0.29 ± 0.20 vs. 0.27 ± 0.18; p = 0.287) [13]. In liver T2WI, liver-to-spleen contrast was marginally yet significantly higher with FSE-DLR than with conventional FSE (0.489 ± 0.090 vs. 0.488 ± 0.090; p < 0.05) [16], whereas liver-to-lesion contrast showed no significant difference (p = 0.48). Therefore, even when statistically significant, DLR-related changes in CR appear to have limited clinical relevance. Overall, DLR likely preserves intrinsic tissue contrast while increasing apparent contrast through denoising and edge enhancement, leading to sharper perceived tissue boundaries due to the synergistic effects of improved SNR and higher effective spatial resolution. Hence, the reader-rated improvement in “Contrast” should be interpreted as enhanced conspicuity rather than an actual alteration in intrinsic tissue signal-intensity ratios.

Regarding artifacts, previous studies have reported inconsistent results. In high-resolution T2WI, the application of DLR incorporating denoising and edge enhancement improved perceived SNR, sharpness, and overall image quality, yet artifact scores significantly worsened [12]. Conversely, other investigations found higher image quality scores, reduced noise, and even fewer artifacts with DLR compared with conventional reconstruction, consistent with our finding of improved perceived noise [14]. DLR is primarily optimized to suppress Gaussian-like random noise; however, it may inadequately address motion-induced ghosting and can make such artifacts appear relatively more conspicuous [2]. Indeed, in head and neck MRI, ghosts overlapping anatomical structures were slightly accentuated after DLR, whereas ghosts occurring outside the anatomy (e.g., within air regions) were reduced [15]. This phenomenon has been attributed to the algorithm’s limited explicit learning of large motion ghosts and to denoising that effectively removes spurious low-signal artifacts outside the body, while edge-oriented interpolation accentuates anatomical boundaries. In breast T2WI, motion artifacts occurring within silicone implants may be accentuated by DLR [12]. In our study, artifact subtypes and their locations (e.g., in-body versus out-of-body ghosting) were not prospectively categorized, and thus the exact distribution of artifact patterns could not be determined. Taken together, the discrepancy we observed between readers for “Artifacts” may partly reflect that our visual assessment did not differentiate artifacts located within anatomical structures from those appearing outside them.

For fat-suppression signal uniformity, DLR did not reduce uniformity and demonstrated a mild tendency toward improvement across readers. DLR primarily contributes to denoising and edge enhancement and has limited capacity to correct the physical spatial inhomogeneity of fat suppression. True uniformity is determined by B0/B1 field homogeneity and by the fat-suppression technique itself; therefore, substantial improvements with DLR alone are not anticipated. Although uniformity was quantitatively evaluated in a head-and-neck MRI study using DLR [15], under a definition that differs from our “visual fat-suppression uniformity,” that investigation reported no significant deterioration, consistent with our finding that uniformity is maintained and may slightly improve in some cases.

This study has several limitations. It was conducted at a single institution using equipment from a single vendor, and external validity remains to be confirmed. Broader and more generalizable evaluations will require inter-vendor comparisons employing standardized assessment criteria. Our study provides preliminary data supporting such future investigations. Furthermore, we focused primarily on image quality and did not directly assess clinical endpoints such as lesion detectability or diagnostic accuracy. Future studies should also evaluate accelerated acquisition protocols enabled by DLR and assess their impact on lesion conspicuity and diagnostic accuracy.

## Conclusions

Using identical raw k-space data, a vendor-provided DLR algorithm combining denoising and super-resolution significantly improved visual image quality and increased SNR in fat-suppressed T2-weighted breast MRI. The change in the CR was minimal, indicating minimal alteration of intrinsic tissue contrast. These findings support DLR as an effective approach for enhancing conspicuity and overall image quality while preserving fundamental tissue contrast relationships.
